# A Combination of Calcination and the Spark Plasma Sintering Method in Multiferroic Ceramic Composite Technology: Effects of Process Temperature and Dwell Time

**DOI:** 10.3390/ma15072524

**Published:** 2022-03-30

**Authors:** Dariusz Bochenek

**Affiliations:** Institute of Materials Engineering, Faculty of Science and Technology, University of Silesia in Katowice, 75 Pułku Piechoty 1a, 41-500 Chorzów, Poland; dariusz.bochenek@us.edu.pl

**Keywords:** multiferroics, ferroelectromagnetic composites, spark plasma sintering, dielectric properties

## Abstract

This study reports a combined technological process that includes synthesis by the calcination powder route and sintering by the Spark Plasma Sintering (SPS) method for multiferroic ceramic composites in order to find the optimal sintering conditions. The effects of temperature on the SPS process and dwell time on the microstructure and dielectric properties of the PF composites were discussed. Research has shown that using the SPS method in the technological process of the multiferroic composites favors the correct densification of powders and allows for obtaining a fine-grained microstructure with good properties and electrophysical parameters in the composite material. The optimal set of parameters and properties is demonstrated by the sample obtained at the temperature of 900 °C for 3 min, i.e., resistivity (6.4 × 10^8^ Ωm), values of the dielectric loss factor (0.016), permittivity at room temperature (753) and permittivity at the phase transition temperature (3290). Moreover, due to the high homogeneity of the microstructure, the strength of the material against electric breakdown increases (when examining the ferroelectric hysteresis loop, the application of a high electric field (3—3.5 kV/mm) is also possible at higher temperatures). In the case of the composite material tested, both the lower and higher temperatures as well as the shorter and longer dwell times (compared to the optimal SPS process conditions) did not contribute to the improvement of the microstructure or the set of usable parameters of the composite materials. The strength of the ceramic samples against electric breakdown has also diminished, while the phenomenon of leakage current increased.

## 1. Introduction

The progress on research into new materials and their impact on the development of material engineering in the world is extremely dynamic, and this applies, inter alia, to improved metal alloys and new types of plastics, ceramics and composite materials for a wide range of applications [1,2,3]. Recently, multiferroics have been interesting materials due to the possibility of connecting their various physical properties into a single component, which increases their potential applications in modern microelectronics, e.g., magnetoelectric transducers and sensors, magnetic data storage, logic, microwave and spintronic devices, multistage information storage devices, etc. [4,5,6]. The group of multiferroics includes ferroelectromagnetic ceramic composites obtained by combining perovskite-type materials (with high ferroelectric properties) and magnetic materials (usually ferrites). In the case of ferroelectromagnetic materials and multiferroic composites, a magnetoelectric effect is desirable [7,8,9]. The modified PbZr_1-x_Ti_x_O_3_ (PZT) materials with perovskite structures have practically been one of the most used in this type of application due to their having the best set of electrophysical parameters (i.e., dielectric, ferroelectric, piezoelectric, electromechanic and optic properties, etc.), and are obtained by various techniques and technological methods [10,11,12]. 

The dynamic growth of demand and the ever-growing requirements for engineering materials simultaneously force the need to develop and improve methods for producing materials with better properties and performance parameters. One of the modern methods of sintering materials is Spark Plasma Sintering (SPS). As an advanced powder metallurgy technology, SPS has been used for the preparation of many kinds of materials, such as ceramics, composites, metals, metallic glass and various amorphous alloys, e.g., [13,14,15]. In the methods of sintering materials with the use of electric current, uniaxial pressure is also applied, while a pulsed current is used to heat the powder’s charge [1,16]. In the SPS method, the sintered powder is heated by Joule heat, which is a result of the impulse current flowing through the punches and graphite dies of the device and through the pressed powder grains, thanks to which the SPS method is characterized by high thermal efficiency. While the current flows through the powder, a spark discharge takes place (at the contact points of the powder grains), thanks to which the absorbed gases and oxides are removed from the surface of the particles, facilitating the formation of active contacts between the sintered powder grains [17,18,19]. 

The technological process of multiferroic composite materials usually uses standard methods of synthesis (solid phase reactions, calcination) and sintering (free sintering, hot pressing), which require the use of high temperatures and long dwell process times. High temperatures promote excessive grain growth, which results in increased porosity and grain size heterogeneity in the microstructure, which often deteriorates the physical properties of the ceramics, e.g., [20,21]. The SPS sintering method has become useful, thanks to the possibility of obtaining materials with a fine-grained microstructure in a much shorter time and at a lower temperature [22] as well as the simultaneously lower energy demand of the sintering process [23,24]. SPS is used for sintering various types of materials: composite materials reinforced with particles and fibers, gradient materials, polymer materials, metal materials (magnetic, amorphous and intermetallic) and ceramic materials (cermets, oxygen and oxygen-free ceramics, superhard materials) [25,26,27]. However, not all electrophysical properties can be improved with the SPS sintering technique for all types of materials. For example, due to the predominance of strong covalent bonds and low diffusion coefficients, ceramic materials are difficult to sinter. Ensuring an appropriate degree of compaction requires the use of a high temperature range. Ferroelectromagnetic ceramic composites also belong to the group of materials that are difficult to obtain (while maintaining high electrophysical parameters). This is due to the deterioration of their ferroelectric and piezoelectric properties as well as the rapid increase in their electrical conductivity as a result of the negative impact of ferrite material. The use of combined methods in the technology of multiferroic composites is not yet widely adopted nor thoroughly analyzed. In one of the works [28], the authors compared the effect of the sintering method (using the SPS method) on the final properties of PFN–ferrite composites. However, the applied SPS method did not significantly improve the parameters of the PFN–ferrite composite, and its dielectric properties deteriorated significantly (with a strong blurring of the phase transition). Thus, as in the classic methods of sintering this type of material, the selection of SPS sintering process parameters should also be selected individually. 

In this paper, the multiferroic PF ceramic composites were obtained by a combined technological process, i.e., synthesis by the calcination powder route and sintering by the Spark Plasma Sintering (SPS) method. The ferroelectric component of the PF composite was PLZT material with the chemical composition Pb_0.98_La_0.02_(Zr_0.90_Ti_0.10_)_0.995_O_3_ (P), while the magnetic component was nickel–zinc ferrite Ni_0.64_Zn_0.36_Fe_2_O_4_ (F), in a proportion of 90/10, respectively. In order to determine the influence of the process temperature on the electrophysical properties of the ceramic PF composites, the SPS process was carried out in a temperature range from 850 to 950 °C (t-series), while the research on the influence of the dwell time on the above properties was carried out in the range of 2 to 5 min (d/t-series). 

## 2. Materials and Methods

### 2.1. Research Material 

The technological process of the multiferroic PF composite samples consisted of several main parts, i.e., synthesis of the ferroelectric material Pb_0.98_La_0.02_(Zr_0.90_Ti_0.10_)_0.995_O_3_ (PLZT), synthesis of the magnetic ferrite material Ni_0.64_Zn_0.36_Fe_2_O_4_ (F), combination of the composite components (sintering of the composite materials) and final treatment of the PF composite samples.

### 2.2. Synthesis 

First, the PLZT ceramic powder was obtained from the mixture’s starting materials, which were homogenized in a planetary mill, dried and calcined at 900 °C for 2 h. A detailed description of the PLZT process is presented in previous work [20]. The ferrite powder (F) was obtained from oxides NiO (99.99% Aldrich, Steinheim, Germany), Fe_2_O_3_ (99.98% Sigma-Aldrich, St. Louis, MO, USA) and ZnO (98% Fluka, Neu-Ulm, Germany). The constituent powders were mixed with a Fritsch planetary ball mill for 8 h (by the wet method) and next calcined at 1100 °C for 4 h. The combination of ferroelectric and magnetic materials to form a ceramic composite was made in proportions of 90% and 10%, respectively. After weighing out the composite components in the correct proportions, the powders were mixed using a Fritsch planetary ball mill for 15 h (by the wet method) and then synthesized by the calcination route at 1000 °C/4 h.

### 2.3. Spark Plasma Sintering (SPS)

The sintering process of the PF composite powders was prepared by the Spark Plasma Sintering (SPS) method using an SPS machine manufactured by the FCT System GmbH, model HP D5 [29], with different temperatures and dwell times used for the SPS process. The constant conditions of the SPS process were: pressure (50 MPa), atmosphere (high-purity argon gas) and heating rate (50 °C/min), while the process temperature and dwell time were variable parameters that ranged from 850 to 950 °C and from 2 to 5 min, respectively. In the above manner, two series of the PF composite samples were obtained depending on the dwell time (d/t-series) and depending on the temperature (t-series). In the first case, four samples were obtained at the following conditions: 900 °C/2 min (PF-A), 900 °C/3 min (PF-B), 900 °C/4 min (PF-C) and 900 °C/5 min (PF-D)—Table 1. In the second case, three samples were obtained at the following conditions: one using a lower temperature of 850 °C/3 min (PF-B^L^), and two using the higher temperatures of 900 °C/3 min (PF-B) and 950 °C/3 min (PF-B^H^)—Table 2. The pressing force (4 kN) is applied uniformly to the die punch during the SPS process. 

**Figure 1:**
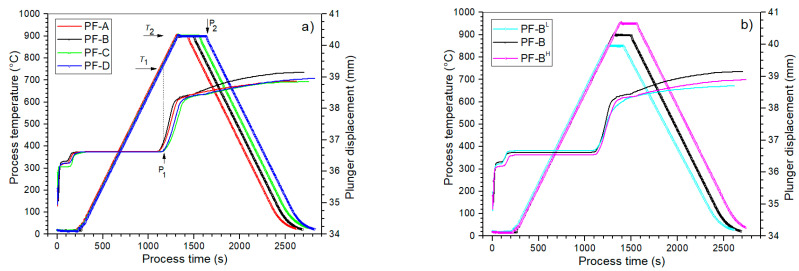
Plots of the SPS process’s temperature rise and plunger displacement for (**a**) d/t-series and (**b**) t-series of the PF composite samples.

The more precisely applied SPS method is also called the Spark Plasma Sintering (SPS)/Field Assisted Sintering Technology (FAST) technique, and is a combination of uniaxial external pressure and electric current in order to rapidly sinter a wide range of powders. A more detailed description of the SPS/FAST mechanism and the physical phenomena occurring during the SPS process, as well as a description of the construction and operation of the device used in this experiment, are presented in detail in work [29]. During the SPS process, a number of its parameters are recorded (temperature, time, heating and cooling rates, pressure force), and the changes in the resistance of the sintered material are visualized. The change in the position of the piston is recorded, which allows for the determination of the compaction curve based on the current pressing pressure and temperature. Thanks to this, the formation of a new phase (with a different specific volume) is registered by changing the position of the piston. Figure 1 shows a schematic course of the SPS process for PF multiferroic ceramic composites (with temperature change and the shift of the die plunger depending on the duration of the process) for two series of samples: (a) d/t-series and (b) t-series. 

### 2.4. Characterization

Composite ceramic pellets with a thickness of 1.0 mm and a diameter of 10 mm were prepared, and mechanical stress was removed by the annealing method at 750 °C/15 min. For electrical measurements, the surfaces of the pellets were covered with a silver electrode (the firing method). 

The SEM microstructure analysis of the composite samples was performed using the scanning electron microscope JEOL JSM–7100F TTL LV (Jeol Ltd., Tokyo, Japan). SEM images were captured using the SB technique (detection of the signals from the secondary and backscattered electron detectors), as well as the BSE technique (detection of backscattered electrons). The chemical composition tests of the ceramic samples were made using an Energy Dispersive Spectrometer (EDS) and Electron Probe Microanalysis (EPMA)—Jeol Ltd., Tokyo, Japan. The SEM, EDS and EPMA tests were performed by using an accelerating voltage of 15 kV, and the Au sputtering technique was used on the sample surfaces (Smart Coater DII-29030SCTR, Jeol Ltd., Tokyo, Japan).

Temperature dielectric properties of the ceramic samples were measured using QuadTech 1920 Precision LCR Meter (Maynard, MA, USA), in temperature ranges from room temperature (*RT*) to 450 °C and at measurement field frequencies from 20 Hz to 100 kHz. DC conductivity tests were performed using the high-resistance meter Keithley 6517B (Cleveland, OH, USA) in temperature ranges from *RT* to 400 °C. Ferroelectric hysteresis loops (*P*–*E*) were examined at a frequency of 5 Hz using a Sawyer–Tower circuit and a Matsusada Inc. HEOPS–5B6 precision high-voltage amplifier (Kusatsu, Japan). The data were stored on a computer disc using an A/D, D/A transducer digital card (National Instrumental Austin, TX, USA) and the LabView computer program. 

## 3. Results and Discussions

### 3.1. SPS Process Flow Analysis 

The start of the densification of the ceramic powder begins at the point P_1_, which corresponds to the temperature *T*_1_ of the initiation of the exothermic sintering reaction (powder densification), which causes a larger sinter shrinkage and an increase in the packing of particles (Figure 1). At the maximum assumed temperature of the process (*T*_2_), the densification rate is reduced, and its completion occurs at point P_2_, that is after the predetermined dwell time at the maximum temperature *T*_2_. When analyzing a series of samples depending on the process time (d/t-series), the highest densification of the PF composite powder occurs in the temperature range from 714 to 900 °C (points P_1_–P_2_), where the largest stroke of the die punch displacement takes place (Figure 1a). The increased dwell time in the SPS process (i.e., the dwell time at the maximum process temperature) does not cause a significant increase in the sinter densification (the plunger displacement in this area does not increase significantly). 

In the case of the second series of the PF composite samples (t-series), i.e., depending on the temperature of the SPS process, the densification in the sintering process initiation area increases (the highest for the PF-B^H^ sample), which is characterized by the greatest displacement of the piston (Figure 1b). As a result of the heating at different temperatures, there is a change in the slope of the densification curves of the ceramic composite materials. In the case of the PF-B^L^ sample (with the lowest sintering temperature), a change in the slope of the densification curve is observed in the form of a marked flattening of the curve. This indicates that too little energy is supplied to the zone with material that is subjected to sintering for the proper course of sintering (correct compaction). 

The first step in determining the optimal technological conditions of the PF composite materials is to calculate the density of individual samples for two series, namely, the t-series and d/t series (Table 1 and Table 2). This part of the analysis showed that, in the case of the tested ceramic samples, the optimal sintering conditions in the SPS process are 900 °C/3 min (PF-B). Under optimal SPS process conditions, a higher density of the PF composite material is achieved compared to the classical technology [20]. 

### 3.2. Crystal Structure and Microstructure 

X-ray powder diffraction patterns of the PLZT, ferrite and PF composite materials were presented in previous works [20] (measurements not presented here), and showed a perovskite structure with a rhombohedral ferroelectric phase and an R3*c* space group for PLZT materials (matching the results for the pattern JCPDS#77-1194), while the spinel structure (F*d*$\bar{3}$*m*) and the best-matched results for the pattern JCPDS#01-077-9718 were shown for ferrite materials (Ni_0.64_Zn_0.36_Fe_2_O_4_). The XRD test of the PF composite powder showed strong peaks derived from the PLZT component and weak ones derived from the ferrite materials, and also confirmed the absence of foreign phases. 

**Figure 2:**
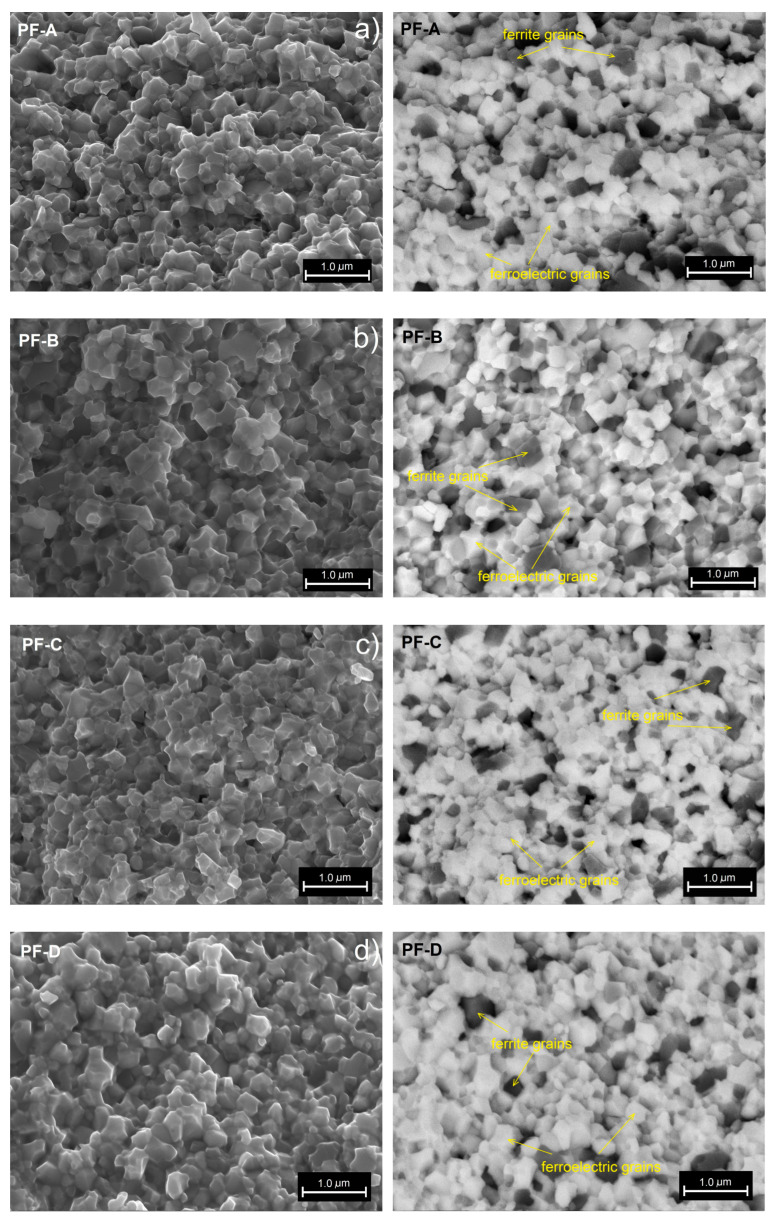
SEM images of the microstructure fracture of the PF composite samples depending on the dwell time (d/t-series): PF-A (**a**), PF-B (**b**), PF-C (**c**), PF-D (**d**), respectively. SB images on the left side, BSE images on the right side.

Figure 2 shows the results of SEM microstructure tests of PF ceramic composite samples obtained for variable times of the SPS process (d/t-series). SEM images were obtained by two image capture techniques, namely (i) the SB technique, i.e., detection of the signals from the secondary and backscattered electron detectors (images on the left side), and the (ii) BSE technique, i.e., detection of backscattered electrons (images on the right side).

For all PF composite samples, a fine-grained microstructure is obtained with closely and strongly connected grains. In the SPS process, the grains of both the ferroelectric and the magnetic components of the PF composite materials are properly crystallized, but areas are also observed where the inter-grain boundaries show an irregular and indistinct shape. SBE imaging was helpful to observe the distribution of the magnetic and ferroelectric components in the composite sample volume. The characteristic distribution of the magnetic component grains (dark areas) in the composite matrix that constitute a component of the ferroelectric material (bright areas) is observed in the entire sample volume. The grains of the ferroelectric component (with a greater regularity of shape) surround the grains of the magnetic component with increased grain size heterogeneity. Comparing the effect of the dwell time at a predetermined constant temperature, it can be concluded that increased SPS process times result in an increase in the grain size in the microstructure of the samples, but the increase is not significant. Important information from the SEM analysis for the d/t series is the achievement of high homogeneity for the microstructure (including the high uniformity of the magnetic grain distribution in the ferroelectric matrix of the PF composite material), which is obtained for optimal SPS sintering conditions (900 °C/3 h). 

**Figure 3:**
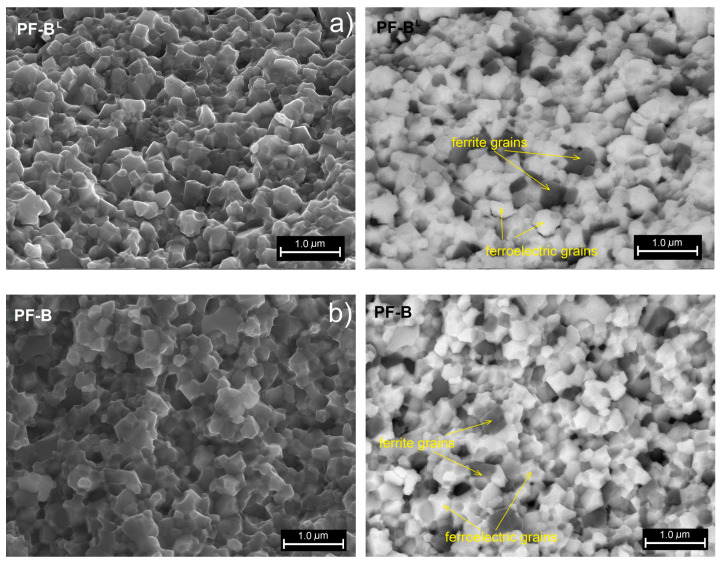

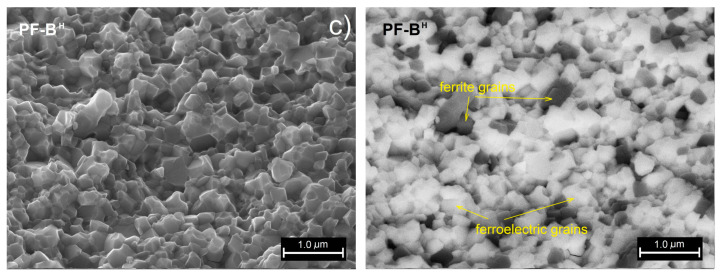
SEM images of the microstructure fracture of the PF composite samples depending on the process temperature (t-series): PF-B^L^ (**a**), PF-B (**b**), PF-B^H^ (**c**), respectively. SB image on the left side, BSE image on the right side.

In the case of the PF ceramic composite series obtained by the variable temperature of the SPS process (t-series), the microstructure tests are presented in Figure 3, i.e., SEM images with the SB technique (on the left side) and the BSE technique (on the right side). As in the previous series, a fine-grained microstructure is obtained for the PF composite samples with strongly connected grains. However, when the temperature of the SPS process changes, the change in the appearance of the microstructure is more pronounced. In the case of too low (PF-B^L^) and too high (PF-B^H^) temperatures in the SPS process, the irregular grain growth of the ceramic powder occurs. In both too low and too high SPS sintering temperatures, the fine grains of the ceramic powder grow unevenly, which increases the grain size heterogeneity in the microstructure of the PF composite samples. The grains of the ferroelectric component surround the grains of the magnetic component, the irregular growth of which is clearly greater. Moreover, there is a tendency to group the magnetic component grains (dark areas in the BSE images) into larger regions with more numerous grains (magnetic grain clusters). This can also cause uneven grain growth in the ferroelectric and magnetic components during the sintering process. 

The sintering temperature has a huge impact on the appearance of the microstructure of the PF composite samples, which is particularly visible for the composite materials obtained by the classical technology [20]. The high temperatures of classical sintering of multiferroic composites contribute to a large disproportion of the growth of ferrite and ferroelectric grains, and consequently result in the high heterogeneity of the microstructure grains. The lower temperatures and shorter dwell times of the SPS process eliminate this undesirable feature of classical technology, limiting the uncontrolled grain growth of the magnetic component of the composite. For a PF ceramic composite, the most optimal sintering temperature is 900 °C, performed at constant process parameters (i.e., sintering time: 3 min; pressure: 50 MPa; atmosphere: argon; heating speed: 50°/min). 

### 3.3. EDS and EPMA Tests 

The quantitative and qualitative EDS analysis method was used to investigate the chemical composition of the PF ceramic composites. The averaged results of the EDS analysis (for five measurements from randomly selected micro-areas on the fractures of the samples) are summarized in Table 3 and Table 4. The EDS microanalysis investigating the materials qualitatively confirmed the assumed share of the individual components of the PF composite samples, viz., lead, lanthanum, zirconium, titanium, nickel, zinc and iron. At the same time, EDS analysis showed no presence of foreign elements. 

The EDS analysis of the percentage share of chemical elements showed slight differences in the chemical composition in the case of the ceramic samples obtained under different SPS process conditions (Table 3 and Table 4). In the series of PF composite samples, the most stable behavior of chemical elements was shown in the PF-B sample (900 °C/3 min). The increasing (or decreasing) dwell time or temperature of the SPS process causes greater discrepancies than the theoretical percentages of its elements. However, all deviations between the theoretical content of chemical elements in the tested PF composite samples and their real content are within the acceptable range. This confirms the high stability of the sintering conditions for the compact in the SPS method. 

**Figure 4:**
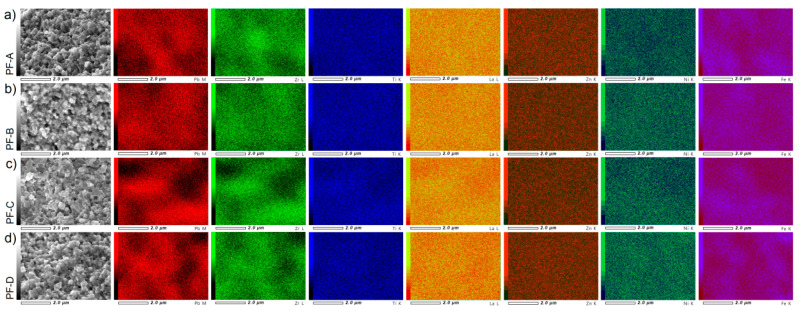
EPMA maps of the PF composite samples (d/t-series): PF-A (**a**), PF-B (**b**), PF-C (**c**) and PF-D (**d**), respectively.

The Electron Probe Microanalysis (EPMA) maps of the distribution of the individual elements for the PF ceramic composites (d/t-series) are displayed in Figure 4. For all analyses of the composite samples, a homogeneous distribution of the individual elements in the surface area is preserved (i.e., the surface obtained after the fracturing of the samples). Places in the microstructure with a smaller amount of the magnetic or ferroelectric component are registered as areas of lower saturation. In the case of larger inequalities in the sample’s surface topography, reading the probe was difficult, which is visible in the darker (shaded) areas on the EPMA maps. 

**Figure 5:**
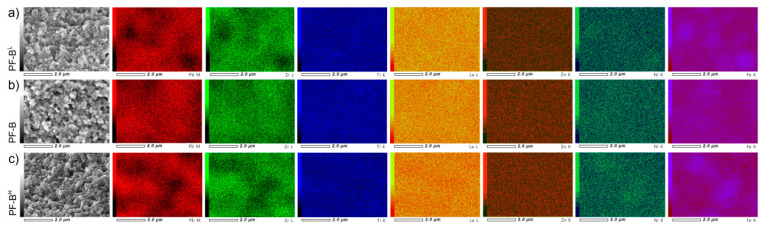
EPMA maps of the PF composite samples (t-series): PF-B^L^ (**a**), PF-B (**b**) and PF-B^H^ (**c**), respectively.

Similar to the d/t-series, the EPMA maps of the PF ceramic composites for the t-series (Figure 5) show that a homogeneous distribution of the individual elements in the surface area of research is preserved. The EPMA study confirmed that the SPS sintering process for ceramic composite powders maintains an even distribution of the constituent elements throughout the sample volume. 

### 3.4. Dielectric Properties 

**Figure 6:**
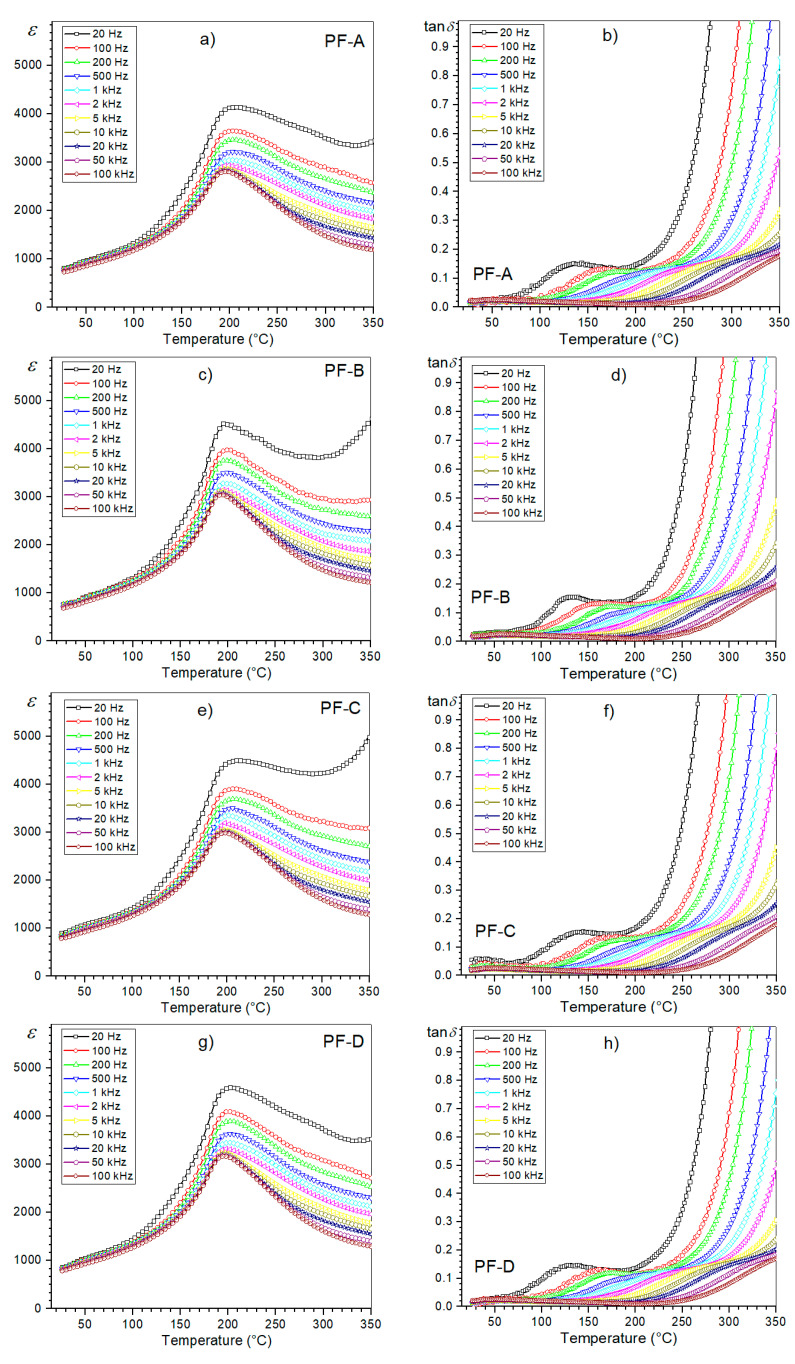
Temperature dependencies of the dielectric parameters, i.e., permittivity and the dielectric loss factor, for the PF composite samples (d/t-series): PF-A (**a**,**b**), PF-B (**c**,**d**), PF-C (**e**,**f**) and PF-D (**g**,**h**), respectively.

At room temperature, the PF composite samples of the d/t-series have average values of electric permittivity of 724 (PF-A), 753 (PF-B), 782 (PF-C) and 795 (PF-D), while at the phase transition temperature (*T_m_*), they are: 3045, 3290, 3350 and 3445, respectively. At *RT*, the dielectric loss factor values range from 0.015 (PF-D) to 0.018 (PF-A). Figure 6 shows the temperature tests of the dielectric properties (permittivity and the dielectric loss tangent) for the d/t-series. The d/t-series composite samples have average values of permittivity with a phase transition diffusion, which is characteristic for multiferroic ceramic composites but also for many other ceramics, e.g., [20,30,31]. However, for this type of composite ceramic material, the determination of the optimal conditions is extremely important due to the danger of deteriorations in its dielectric properties. In addition to a significant reduction in the value of electric permittivity, the phase transition blur may increase significantly, deteriorating the final properties of the multi-grained composite [28]. In this type of composite compound, it is also typical that the dielectric loss values remain low in the ferroelectric phase, but are much higher compared to the ferroelectric component of the composite materials (PLZT material in this case). The conducted measurements of the dielectric properties did not show any significant changes in the temperature plots of permittivity ε(*T*) and the dielectric loss tangent tan *δ*(*T*) with the change in the SPS process time. 

**Figure 7:**
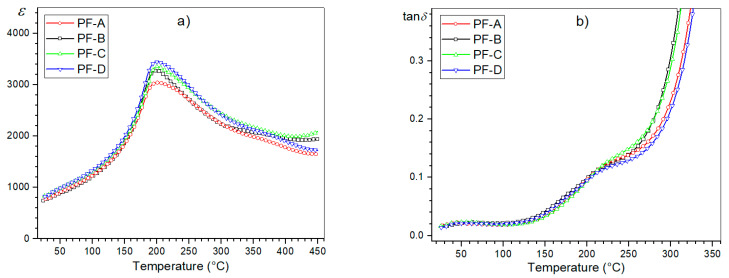
Temperature dependencies of the dielectric parameters for the PF composite samples (d/t-series): permittivity (**a**) and the dielectric loss factor (**b**), for 1 kHz.

However, slight differences are visible in the values of the maximum permittivity ε_m_ at the phase transition temperature *T*_m_. With the increase in the process time, the values of ε_m_ are higher, which is presented in the summary plot of ε(*T*) for 1 kHz (Figure 7). A longer SPS process time favors the grain growth of the ceramic sample. In the case of the ceramic materials, one of the factors influencing their dielectric properties may be related to the microstructure of the ceramic sample (more precisely, the grain size). The research in this direction, presented for example in [32], showed that ceramic materials with larger grains in their microstructure have higher values of permittivity. In this work [32], three models have been proposed to account for the grain-size dependence of ceramic materials. Based on the PbO grain boundary model [32,33], when the grain size decreases, the volume fractions of the grain boundaries increase. As a consequence of this, the dielectric constant values of the ceramic samples decrease. Based on the defect model [32,34] the grain boundaries increase as the grain size decreases (increasing the number of the lattice defects), which contributes to the decrease in the dielectric constant. Because the ferroelectric domain’s structure is formed close to the Curie temperature, lattice distortion energy is released by the domain’s formation [32].

Temperature dielectric tests also showed that the PF-B sample (900 °C/3 min) shows the phase transition in a narrower range of temperatures, which may indicate optimal technological conditions for this type of composite material. The diffused nature of the phase transition in the perovskite-type materials can increase due to the non-uniform element distribution in the units cell of the crystal structure [35]. This may be related to the appearance of composition fluctuations (a disturbance in the distribution of ions at the B site in the perovskite unit cell), causing the occurrence of micro-areas with different local Curie temperatures [36,37]. The various admixtures can also induce the formation of non-identical units cell, and the diffused nature of the phase transition can arise due to the coexistence of two different phases in the samples over a wide range of temperatures [38].

In the case of the tan *δ*(*T*) graphs, the temperature changes of the dielectric loss tangent values do not show significant deviations in the ferroelectric phase of the d/t-series for the composite samples (Figure 7b). Slight differences in the value of the dielectric loss tangent occur above the phase transition in composite samples (e.g., for 250 °C, tan δ values in the range of 0.1281 to 0.1476). Generally, in the case of ceramic materials with increases in temperature, the dielectric loss increases rapidly. At higher temperatures, the mobility of the charge carriers increases (the polarization increases), which leads to a high dielectric loss (the effect of charge accumulation at the grain boundaries) [39]. In the PF composite samples, the dielectric loss tangent values remain relatively low, but in comparison with the PLZT ceramic samples, they are still much higher (the negative effect of the addition of ferrite) [20].

**Figure 8:**
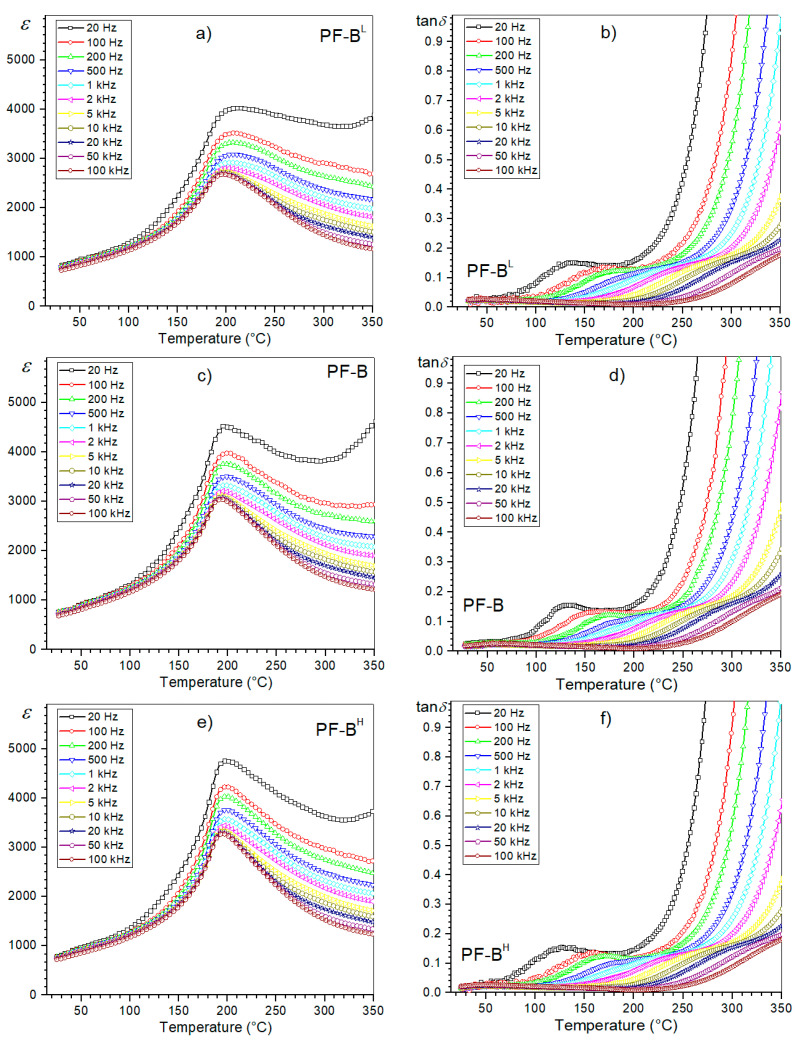
Temperature dependencies of the dielectric parameters, i.e., permittivity and dielectric loss factor, for the PF composite samples (t-series): PF-B^L^ (**a**,**b**), PF-B (**c**,**d**) and PF-B^H^ (**e**,**f**), respectively.

**Figure 9:**
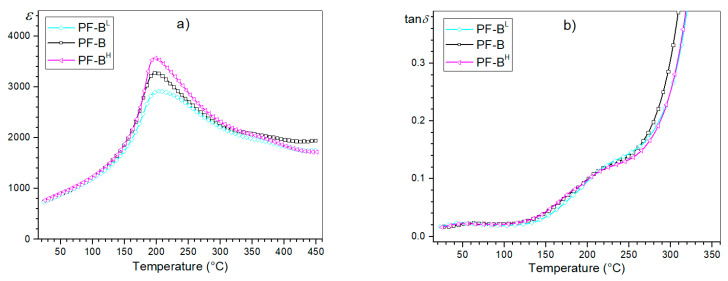
Temperature dependencies of the dielectric parameters for the PF composite samples (t-series): permittivity (**a**) and the dielectric loss factor (**b**), for 1 kHz.

At *RT*, the composite samples of the t-series (Figure 8) also have average values of electric permittivity of 740 (PF-B^L^), 753 (PF-B) and 765 (PF-B^H^), while at the *T_m_*, they are 2920, 3290 and 3570, respectively. At *RT*, the dielectric loss factor values range from 0.016 to 0.017. As for the d/t-series, the temperature curves of *ε*(*T*) and tan *δ*(*T*) for the t-series have a similar appearance (Figure 9). However, in the case of analyzing the influence of the temperature of the SPS process on the dielectric properties of the composite samples, the changes are more pronounced than in the case of the d/t-series (Figure 7). Providing more energy to the zone of the material subjected to sintering (in the form of heat energy) promotes grain growth. This contributes to an increase in the maximum value of permittivity for the composite samples [32,40]. 

### 3.5. DC Electric Conductivity 

**Figure 10:**
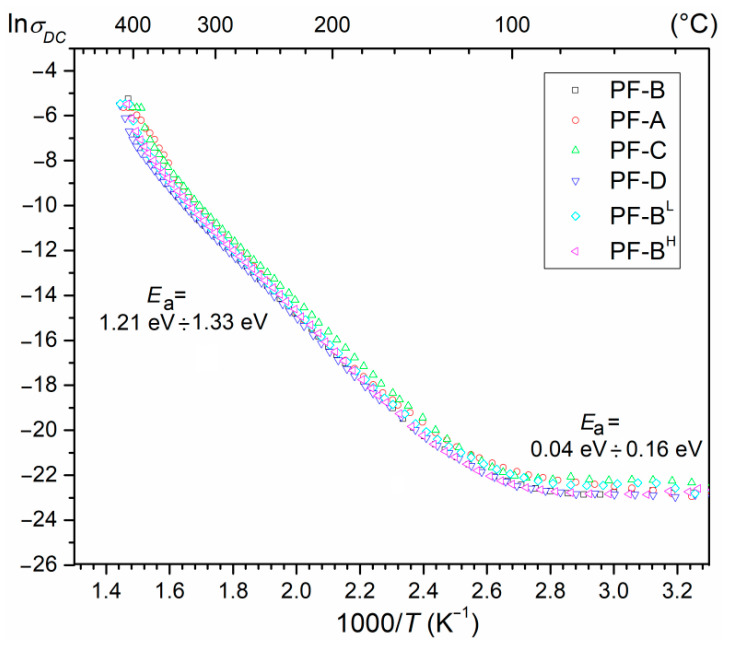
The ln*σ*_DC_ (1000/*T*) relationship for the PF composite samples.

At *RT*, the DC resistivity *ρ*_DC_ of the PF ceramic composites is in the range of 2.8 × 10^8^ Ωm (for PF-B^L^) to 6.4 × 10^8^ Ωm (for PF-B). Figure 10 shows the ln *σ_DC_*(1000/*T*) relationship for the series of PF composite samples. Initially, the conductivity changes slightly as the temperature rises. Above 100 °C, the conductivity increases (resulting in the total electrical resistance decreasing and a negative temperature coefficient of resistance (NTCR)), which is typical for insulating materials with n-type conductivity [41]. This behavior could be due to the increase in oxygen vacancies as well as the concentration of electrons [42,43]. Under these conditions, the occurring defects acquire enough energy to exceed the potential barrier, contributing to an increase in electrical conductivity. The value of the activation energy of the PF ceramic composite samples falls within the range of 0.04 to 0.16 eV (for the I region at lower temperatures) and 1.21 to 1.33 eV (for the II region at higher temperatures)—Table 5 and Table 6.

The conductivity at the lower-temperature side is mainly related to the ionization processes and the electrons or holes that are the dominating charge carriers [43]. At the higher temperatures, the activation of the extrinsic defects occurs, and the mobility of these defects is a predominant contributor to the conductivity. However, at very high temperatures, the concentration and movement of the intrinsic defects increase and play a dominant role in the conduction process of ceramic materials [42,43]. 

The electrical conductivity of the PF composite materials could be dominated by electrons or holes depending upon the extrinsic-defect-type structure and on the degree of concentration [44]. The formation of vacancies or defects is a frequently occurring effect of the technological process in perovskite-type ferroelectric materials. At high temperatures in the sintering process, the volatilization of volatile metals generates the cation vacancies in the structure, which may be compensated by the generation of holes. On the other hand, oxygen losses and the formation of oxygen defects (oxygen vacancies) also occur at higher sintering temperatures because of the charge imbalance, resulting in the generation of electrons via the ionization of the oxygen vacancy [45]. 

The ionization vacancies to generate the electrons and holes can be represented by Kröger–Vink notation [43,46]:

(1) $$V_{Pb,\;La}↔V_{Pb,\;La}^{\prime }+h^{.}$$

and

(2) $$V_{O}↔V_{O}^{\prime }+e^{\prime }\;\mathrm{or}\;V_{O}^{\prime }↔V_{O}^{\prime \prime }+e^{\prime }$$

Beside the oxygen vacancies, other thermally induced defects such as stacking faults, Frenkel defect pairs and interfacial charge accumulation can be reduced by annealing [47,48]. These defects are usually created in ceramic materials at high temperatures during grain growth, and proper densification can be obtained using a slow cooling rate, but too slow of a cooling rate can increase the possibility of volatilization of the volatile metals. The formation of vacancies (and their related defects) evokes the deterioration of a number of properties of perovskite ceramic materials [49,50,51]. A high defect concentration leads to an increased leakage current as well as an increase in the electrical conductivity of the ceramic samples, which hinders or even prevents the poling process and *P-E* measurements. This phenomenon was observed in the case of the PF ceramic composites. The available literature [52,53,54] states that defect reduction in this type of ceramic material can be achieved using the proper methods of the technological process, including introducing the proper admixtures, sintering in an appropriate environment, using the high-pressure sintering methods, etc. For example, the use of oxygen atmosphere during the sintering process as well as annealing can result in reductions in the oxygen vacancies [42,43]. On the other hand, a technological process with an argon atmosphere does not lower the concentration of oxygen vacancies, but it may alleviate the existing defects on the outer side of the grain and increase the accumulation of defects at the grain–grain boundary [55,56]. 

### 3.6. Ferroelectric Properties 

**Figure 11:**
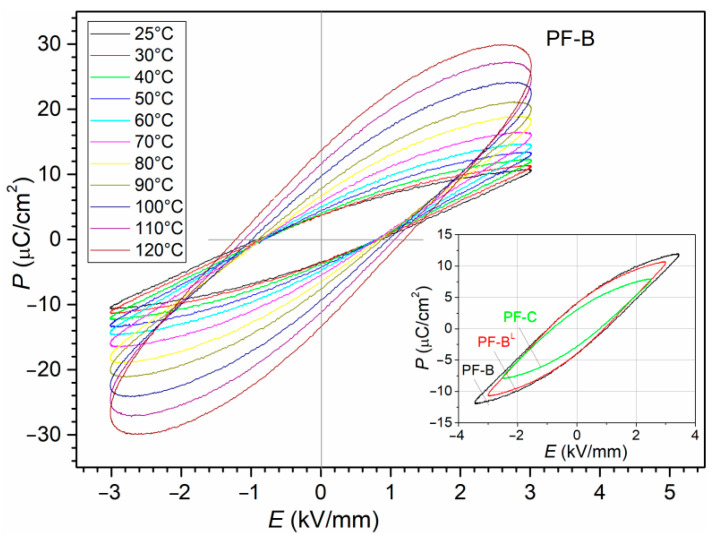
The *P-E* hysteresis loop for the PF composite samples.

The *P-E* temperature tests (Figure 11) of the ferroelectric properties of the PF composite obtained by the SPS method (under various process conditions) could be successfully performed only for the PF-B sample (900 °C/3 min). At *RT*, the sample shows a narrow hysteresis loop (with a coercive field *E*_c_ = 0.87 kV/mm) and a residual polarization of *P*_r_ = 3.67 µC/cm^2^ (for an applied field of 3 kV/mm). With increasing temperatures, the *P*_r_ and *E*_c_ increase (13.73 µC/cm^2^ and 1.21 kV/mm, respectively, at 120 °C), but the hysteresis loop loses its saturation and takes the shape characteristic of linear-type ceramic materials with losses. In the case of the PF-B^L^ (850 °C/3 min) and PF-C (900 °C/4 min) samples, at *RT*, the value of the maximum field applied to the sample (near breakthrough) was 3.0 kV/mm and 2.5 kV/mm, respectively. The remaining samples showed a significantly high electrical conductivity and a relatively high leakage current (even when applying low fields), which made it impossible to carry out *P-E* measurements on the entire series of samples under comparable conditions and at higher temperatures. 

When an electric field is applied to ferroelectric materials, the current response is associated with the linear dielectric response of the ceramics and is due to the switching of the domains [57]. When the applied electric field is low, most of the current contribution comes from the dielectric response of the ceramics. After exceeding a certain value of the electric field, the switching of domains takes place inside the material (the current response due to domain switching starts dominating the total current of the material). The switching in ferroelectric material takes place by domain nucleation and domain wall movement [57]. The presence of oxygen vacancies, and relatedly, high defect concentrations, are a common cause of the increased leakage current and electrical conductivity of the ferroelectric ceramic materials, which makes it difficult (and sometimes impossible) to apply high electric fields to the ceramic sample during the *P-E* measurements as well as the poling process. 

Generally, using the SPS method in the technological process of the PF multiferroic composites favors the correct densification of powders and allows for the obtaining of a fine-grained and homogeneous microstructure with good properties and electrophysical parameters of the PF composite material compared with classical technology [20,58]. Since the SPS method has many process parameters influencing the final properties of the material, their selection is extremely important to optimize the sintering process of a specific type of material. In the study, two variable parameters were selected to determine the optimal sintering conditions of the SPS process: temperature and dwell time. The research conducted on other materials [59] showed that the optimal set of usable parameters can also be obtained by changing the third important parameter of the SPS process, namely the applied pressure. In [59], the authors demonstrated that, with a lower temperature and, at the same time, a higher holding pressure and dwell time, a satisfactory result can be achieved as with a higher temperature and a lower pressure and dwell time (i.e., a similar result can be achieved with different SPS parameters). However, it was not confirmed that there is an optimal combination of parameters which would be suitable for different types of sintered material. For two series of the samples, i.e., sintered at different temperatures (850, 900 and 950 °C) and different dwell times (2, 3, 4 and 5 min), the best results were obtained for the PF composite material sintered at 900 °C within 3 min, i.e., resistivity at *RT* (6.4 × 10^8^ Ωm), values of the dielectric loss factor at *RT* (0.016) and permittivity (753 and 3290) at *RT* and *T_m_*, respectively. 

## 4. Conclusions

This work obtained and examined PF multiferroic ceramic composites (based on ferroelectric and ferrite materials) by combined technological processes (i.e., synthesis by calcination and sintering by Spark Plasma Sintering) and with the optimization of sintering conditions. Two approaches to changing the parameters of the SPS process (temperature and dwell time) were used in the study in order to search for optimal sintering conditions for multiferroic composite materials. 

The SPS method used in the technological process of the PF multiferroic ceramic composites has a positive effect on the densification of powders and the final properties of the composites. PF composite materials sintered by the SPS method are characterized by a fine-grained structure and a high relative density, while maintaining optimal physical properties. On the basis of the conducted tests, the most appropriate SPS process parameters (at which the PF composite material shows an optimal set of parameters) are the following: temperature 900 °C, dwell time 3 min, pressure 50 MPa, an atmosphere of high-purity argon gas and a heating rate 50 °C/min. This appropriate selection of SPS process conditions for PF multiferroic composite samples results in the limiting of the formation of vacancies (reducing the electrical conductivity), and the material maintains good dielectric properties. Moreover, the correct microstructure of the PF sample ensures its high strength against electric breakdown, including at higher temperatures. Research has shown that, in the case of PF composite materials, both lower and higher temperatures, as well as shorter and longer SPS process times (compared to optimal SPS process conditions), do not contribute to the improvement of the microstructure (increases in grain size heterogeneity). These changes do not improve the set of usable parameters for composite materials, and the phenomenon of leakage current increased. 

## Figures and Tables

**Table 1:** Characteristic SPS process parameters of the PF composite samples—dwell time series (d/t-series).

Sample	Process Temperature (°C)	Dwell Time *t* (min)	Pressure (MPa)	Heating Rate (°/min)	Plunger Displacement (mm)	Density (g/cm^3^)
PF-A	900	2	50	50	1.78	6.91
PF-B	900	3	50	50	1.86	7.23
PF-C	900	4	50	50	1.75	6.95
PF-D	900	5	50	50	1.82	6.84

**Table 2:** Characteristic SPS process parameters of the PF composite samples—temperature series (t-series).

Sample	Process Temperature (°C)	Dwell Time *t* (min)	Pressure (MPa)	Heating Rate (°/min)	Plunger Displacement (mm)	Density (g/cm^3^)
PF-B^L^	850	3	50	50	1.54	6.76
PF-B	900	3	50	50	1.86	7.23
PF-B^H^	950	3	50	50	1.82	7.03

**Table 3:** Theoretical and experimental percentages of the individual components of the PF composite samples—dwell time series (d/t-series).

Elements	PF	PF-A	PF-B	PF-C	PF-D
	TH (%)	EXP (%)			
Pb	55.389	55.68	55.45	55.51	55.64
La	0.785	0.88	0.80	0.81	0.82
Zr	22.284	21.70	21.84	21.81	21.72
Ti	1.299	1.15	1.28	1.29	1.26
Zn	0.714	0.76	0.82	0.80	0.78
Ni	1.139	1.31	1.22	1.25	1.26
Fe	3.385	3.43	3.48	3.46	3.48
O	15.33	15.09	15.11	15.07	15.04

**Table 4:** Theoretical and experimental percentages of the individual components of the PF composite samples—temperature series (t-series).

Elements		PF-B^L^	PF-B	PF-B^H^
	TH (%)	EXP (%)		
Pb	55.389	55.74	55.45	55.29
La	0.785	0.73	0.80	0.82
Zr	22.284	21.69	21.84	21.98
Ti	1.299	1.12	1.28	1.22
Zn	0.714	0.71	0.82	0.77
Ni	1.139	1.25	1.22	1.26
Fe	3.385	3.64	3.48	3.67
O	15.33	15.12	15.11	14.99

**Table 5:** Parameters of PF composite samples—dwell time series (d/t-series).

Sample	*ρ*_DC_ (Ωm)	*E*_a_ (eV) I Region/Lower Temperatures/	*E*_a_ (eV) II Region/Higher Temperatures/
PF-A	5.8 × 10^8^	0.16	1.33
PF-B	6.4 × 10^8^	0.04	1.21
PF-C	4.2 × 10^8^	0.03	1.23
PF-D	3.6 × 10^8^	0.08	1.23

**Table 6:** Parameters of PF composite samples—temperature series (t-series).

Sample	*ρ*_DC_ (Ωm)	*E*_a_ (eV) I Region/Lower Temperatures/	*E*_a_ (eV) II Region/Higher Temperatures/
PF-B^L^	2.8 ×10^8^	0.04	1.22
PF-B	6.4 × 10^8^	0.04	1.21
PF-B^H^	6.2 × 10^8^	0.06	1.23

## Data Availability

Data are contained within the article.
